# International lessons in new methods for grading and integrating cost effectiveness evidence into clinical practice guidelines

**DOI:** 10.1186/s12962-017-0063-x

**Published:** 2017-02-10

**Authors:** Kathryn M. Antioch, Michael F. Drummond, Louis W. Niessen, Hindrik Vondeling

**Affiliations:** 1 Guidelines and Economists Network International (GENI), Department of Epidemiology and Preventive Medicine, Monash University Australia, 27 Monaro Road, Kooyong, VIC 3144 Australia; 2 GENI Board, Centre for Health Economics, University of York, Alcuin A Block, Heslington, York, YO10 5DD UK; 3 GENI Board, Warwick Medical School, Liverpool School of Tropical Medicine, Pembroke Place, Liverpool, L3 5QA UK; 4 GENI Board, Center for Health Economic Research, Centre for Applied Health Services Research, J.B. Winsløws Vej 9, Indgang B, 1. Sal, 5000 Odense C, Denmark

**Keywords:** I1: health, D61: cost benefit analysis, I180: Health-Government policy, Regulation, Public health, O320: management of technological innovation and R&D

## Abstract

Economic evidence is influential in health technology assessment world-wide. Clinical Practice Guidelines (CPG) can enable economists to include economic information on health care provision. Application of economic evidence in CPGs, and its integration into clinical practice and national decision making is hampered by objections from professions, paucity of economic evidence or lack of policy commitment. The use of state-of-art economic methodologies will improve this. Economic evidence can be graded by ‘checklists’ to establish the best evidence for decision making given methodological rigor. New economic evaluation checklists, Multi-Criteria Decision Analyses (MCDA) and other decision criteria enable health economists to impact on decision making world-wide. We analyse the methodologies for integrating economic evidence into CPG agencies globally, including the Agency of Health Research and Quality (AHRQ) in the USA, National Health and Medical Research Council (NHMRC) and Australian political reforms. The Guidelines and Economists Network International (GENI) Board members from Australia, UK, Canada and Denmark presented the findings at the conference of the International Health Economists Association (IHEA) and we report conclusions and developments since. The Consolidated Guidelines for the Reporting of Economic Evaluations (CHEERS) 24 item check list can be used by AHRQ, NHMRC, other CPG and health organisations, in conjunction with the Drummond ten-point check list and a questionnaire that scores that checklist for grading studies, when assessing economic evidence. Cost-effectiveness Analysis (CEA) thresholds, opportunity cost and willingness-to-pay (WTP) are crucial issues for decision rules in CEA generally, including end-of-life therapies. Limitations of inter-rater reliability in checklists can be addressed by including more than one assessor to reach a consensus, especially when impacting on treatment decisions. We identify priority areas to generate economic evidence for CPGs by NHMRC, AHRQ, and other agencies. The evidence may cover demand for care issues such as involved time, logistics, innovation price, price sensitivity, substitutes and complements, WTP, absenteeism and presentism. Supply issues may include economies of scale, efficiency changes, and return on investment. Involved equity and efficiency measures may include cost-of-illness, disease burden, quality-of-life, budget impact, cost-effective ratios, net benefits and disparities in access and outcomes. Priority setting remains essential and trade-off decisions between policy criteria can be based on MCDA, both in evidence based clinical medicine and in health planning.

**Electronic supplementary material:**

The online version of this article (doi:10.1186/s12962-017-0063-x) contains supplementary material, which is available to authorized users.

## Background

Economic evidence is becoming influential in health technology assessment (HTA) world-wide. However, most health care practices and procedures are not subjected to HTAs, thereby limiting the impact of economic evidence. CPGs offer the potential for economists to be centrally involved in increasing the cost-effectiveness of health care provision. However, examples of the use of economic evidence in such guidelines, and the subsequent integration of such evidence into clinical practice and national decision making are limited. This may be because of resistance from the professions, paucity of economic evidence or lack of policy commitment.

Important components of the evidence used in the development of CPGs include the use of systematic reviews of the literature, decision rules, WTP, opportunity costs, and end of life/social value judgement along with techniques to grade the economic evidence. Such evidence can be graded by guidelines or ‘checklists’ to establish which evidence should be used to inform decisions based on the rigor of the methodology. New developments in all of these areas are cutting edge techniques that provide a platform for improved contribution by health economists in decision making world-wide and are considered in this paper. The principal purpose of this paper is to address the implications of these methodologies for the integration of economic evidence into CPG agencies world-wide and their implications for Australian reforms by the Parliament, governments and health services. These issues were discussed by Board members of the Guidelines and Economists Network International^1^ (GENI) in presentations at the 9th IHEA World Congress.

The GENI is an international association which enables health economists, epidemiologists, clinicians and medical experts world-wide to work with prominent international bodies, health services and governments. GENI’s agenda is to facilitate the effective integration of CPGs, economic and clinical evidence into national decision making and clinical practice in the health sector. GENI aims to forge linkages with bodies that set the standards for appropriate treatment under different conditions that may link to contracts or regulatory processes such as insurers and national government funding systems. Linkages to Evidence Based Medicine (EBM), service delivery and related government regulatory and funding systems are central. GENI’s has twelve international board members and a large twenty-nine member management team comprising CEO, Medical Advisory Committee, Directors, senior managers and researchers, with over 830 GENI LinkedIn Group affiliates.

At the 9th IHEA Congress, five of GENI’s Board members from America, UK, Australia, Canada and Denmark discussed new methodologies and economic guidelines or ‘checklists’ for incorporating economic evidence into CPGs, clinical practice and national decision making. The session considered guidelines recently developed for the conduct and reporting of economic evaluations. These guidelines assess studies for decision-making in technology assessment for reimbursement or developing CPGs. The session also discussed use of CEA evidence in CPG processes in Australia by NHMRC, three Local Hospital Networks (LHNs) in Victoria and implications for related national and state health policy reforms led by the Council of Australian Governments (COAG) and reviewed by the Australian Parliament. An impetus for the international discussion at IHEA was a request from the NHMRC for input from GENI about the Council’s review of its processes to develop and approve guidelines in Australia using the costs and benefits evidence and also socio-economic evidence. The focus on these evidence based medicine (EBM) approaches involving CPGs and CEA evidence has also been an important feature in some reforms by the Australian Parliament, Governments and health services. The methodologies discussed in this paper may further assist this reform agenda, along with the deliberations of the NHMRC and other regulatory bodies that approve CPGs world-wide.

Firstly, we discuss Australian health policy reforms in Commonwealth-State health agreements involving, *inter alia*, national implementation of LHN governance structures and hospital Activity Based Funding (ABF) based on the Victorian model. Recommendations were provided to COAG and the Senate based on Victorian evaluations of applications of NHMRC’s CPG methods using CEA in Victorian LHNs and the risk adjustment of ABF, given relevance to legislation for health agreements and related authorities. The Senate also considered reforms for palliative care, rural health and the Aged Care Quality Agency and recommendations were submitted to the Senate about crucial CPG and CEA issues. COAG’s 2016 Heads of Agreements on Public Hospital Funding will reduce ABF for specified adverse events, ineffective or harmful treatments and readmissions. Recommendations to the Senate about risk adjusting ABF and measurements of quality outcomes and disseminating NHMRC’s EBM methods Australia-wide are also discussed. The NHMRC’s CPG development process review is then considered.

That is followed by consideration of the CHEERS Task Force which has consolidated guidelines for assessing health economic evaluation studies and recently published a consensus statement, along with the checklist. Recent developments for MCDA that broaden the objectives of evaluation in health care are considered along with the International Society for Pharmacoeconomics and Outcomes Research’s (ISPOR) MCDA Emerging Good Practices Task Force that has developed a common definition for MCDA in health care and good practice guidelines for conducting MCDA. The Hopkins Review is then discussed which examines if and how economic evidence impacts on health policy, including clinical guidelines, in the USA. It also examines the strengths and weaknesses of existing checklists used to assess best practices in economic evaluations. The review of studies also addresses the use of economic outcomes in policy and decision making. Finally, we conclude with implications of these deliberations world-wide for health services, governments and agencies that develop CPGs such as National Institute of Health and Care Excellence (NICE), US Guidelines clearing house, NHMRC and the USA Centre for Disease Control and Prevention (CDC).

## Review

### Lessons for Australian parliament, National Health and Medical Research Council, Governments and Health services

#### Australian Commonwealth-State Health agreements and reforms

In Australia, there are several agreements between Commonwealth and State governments through the Council of Australian Governments (COAG), that specify health policy and reform directions, including the National Healthcare Agreement (NHA), the National Health Reform Agreement (NHRA), National Partnership Agreements and the Intergovernmental Agreement on Federal Financial Relations. Further, the April 2016 Heads of Agreement on Public Hospital Funding forms the basis of negotiations leading towards a time-limited addendum of the NHRA, in the form of an additional schedule, to commence on 1 July 2017.

This suite of Agreements is designed to set out the architecture for a nationally unified and locally controlled health system. They also encourage improved quality of care and the cost-effective use of guidelines, clinical pathways and other EBM initiatives [1].

Antioch briefed COAG during 2008, 2009 and 2010 about the new health agreements. She also briefed the Australian Senate from 2010 to 2013 on COAG Agreements and related authorities and, in 2016, on new COAG Heads of Agreements. Some briefs included evidence that implementing NHMRC methodologies for CPG implementation with economic and clinical evidence in Victorian LHNs resulted in cost effective service provision in the context of ABF. She recommended the development of *State Centres of EBM Health Services and Workforce Redesign*, use of NHMRC and international methods with economic and clinical evidence. an International Centre of EBM and Health Economics and the need for adequate risk adjustment of ABF [2–4].

Antioch’s [2] COAG brief addressed a reform agenda for the 2009 Australian Healthcare Agreements (AHA) re-negotiation involving two areas. Firstly, she proposed new state centres to integrate the economic and clinical evidence and CPGs into clinical practice using methods by the NHMRC [5–9], Antioch et al. [10], Drummond et al. [11] and organisations such as GENI. Secondly, she argued the equity of the AHCA formulae and index can be improved using risk adjustment to align funding with health need by using either Diagnostic Cost Group-Hierarchical Condition Categories (DCG-HCC) relative risk scores or Diagnostic Related Group (DRG) cost weights, Evidence she published in European Journal of Health Economics concerning risk adjustment requirements for the ABF formulae in Victoria relating to severity markers for state-wide referral services e.g. transplantation, major trauma was also referenced [2, 12].

Antioch’s [3] COAG brief outlined national cost savings of $273.524 m pa (2006 prices) and $1367.620 m over five years via implementation of the proposed State Centres, modelled on cost savings achieved by reductions of adverse events and length of stay in the Victorian hospital experience. That brief attached advice from the Northern Territory (NT) Health Minister who expressed interest in risk adjustment for ABF implementation given the demographic and geography of NT are such that the use of a standard Australian profile runs a severe risk of the jurisdiction being disadvantaged [3]. The ABF formulae subsequently developed by the Australian Government included a risk adjustment to the formulae called ‘indigenous adjustment’ which still remains along with other adjustments [13].

Antioch’s COAG brief, which was also published by the Senate Standing Committee on Finance and Public Administration Inquiry into COAG Reforms relating to Health and Hospitals [4], stated that some Health Ministers advised that their Departments were considering her reform proposals for their implementation of the National Health Agreements and Partnership programs, with one large jurisdiction indicating that the State planned to introduce a State Centre similar to her proposed State Centres. The brief also recommended the establishment of an International Centre of Evidence Based Medicine and Health Economics.

Antioch’s COAG and Senate submissions indicated that the ‘State Centres’ recommendation arose from national stakeholder engagement during her 2007 presentations in all Australian States and Territories and NZ about the implementation and evaluation of the initiatives she led in the Victorian LHNs and subsequent liaison with the Federal Health Ministers’ Office. The national presentations were sponsored by the Australian Health Care and Hospital Association and the Women’s and Children’s Hospital Australasia in the context of the renegotiation of National Health Agreements. She provided a Cost Benefit Analysis (CBA) of national implementation of the State Centres in her invited submission to the Senate Inquiry concerning the establishment of the National Health Performance Authority and also the Independent Hospital Pricing Authority which calculated net benefits of $269.6 m per annum (2006 prices) [14, 15]. The CBA was modelled on the evaluation of the Victorian LHN initiatives. Many features of the national reforms by COAG, and under consideration by then Australian Parliament, were based on the Victorian health system including ABF and new hospital network governance through LHNs. Evidence of cost effective EBM implementation in Victoria was of interest.

#### Australian parliament inquiries on health agreement legislation and health policy reforms

Parliamentary Inquiries since 2010 made recommendations about policy and legislation to implement elements of the Health Agreements and other health policy. The Senate Report on the Federal Finance (National Health and Hospitals Network) Bill [16] cited Victorian health system effectiveness and efficiency evidence provided by Antioch [17] in support of the new national arrangements. The Committee recommended that the Senate pass the Bill and recognized the Bill is ‘a vital piece of legislation which will enable the implementation of significant elements of the health and hospital reforms agreed by COAG in April 2010′. Economics Legislation Senate Committee [16]. More recently, various State governments are implementing State Centres to facilitate best practice and innovation in NSW, Victoria and Queensland.^2^ The methodologies in this paper and deliberations of more recent Senate Inquires in 2012, 2013 and 2016 outlined below could assist such State Centres along with Australian governments, health services and the NHMRC.

The Senate Inquiries during 2012 into Palliative Care in Australia and Factors Affecting the Supply of Health Services and Medical Professionals in Rural Areas addressed CPGs and the use of health economics to improve efficiency, outcomes and prioritization of health technologies and service delivery. Stakeholders in the Palliative Care Inquiry advocated urgent updated CPGs for palliative care, advanced care directives, dementia diagnosis, pain management and case management to enable improvements in cost effective service provision and continuum of care. Antioch recommended that CPG updates by COAG, Federal Department of Health and NHMRC could use CPGs by NICE and Agency for Healthcare Research Quality (AHRQ) USA CPG Clearinghouse concerning opioids, advanced care directives/planning and radiotherapy. She advocated that CEA evidence for new Australian CPGs in palliative care should be based on NHMRC methodologies [18]. The Senate report on Palliative Care in Australia recommended that COAG should consider developing and implementing a case management model; and a uniform national palliative care pathway that clarifies when general palliative care moves into specialist palliative care, and maps the diagnosis and referral process to ensure that a palliative patient’s journey involves coordinated access to all necessary services. It also recommended that a national model legislation for advanced care planning should be developed and the NHMRC should report its work on alternative therapy claims in relation to palliative care and [19].

Stakeholders in the 2012 Rural Health Senate Inquiry argued for urgent mechanisms for rapid EBM translation of CEA evidence and CPGs into rural practice with the input of economists. They also advocated improved Evidence Based Planning (EBP) to identify rural supply and demand factors and prioritisation. Antioch [18] recommended that the Federal government undertake a national review of EBM translational work to prevent duplication, with a consolidated and rapid disseminate EBM evidence to rural areas. For EBP she recommended the uptake of evidence concerning disease burden, epidemiological data and CEA of interventions, for demand and supply modelling. Any gaps in the evidence identified in the national review should be addressed by Federal and State governments, NHMRC and all States [20]. A key Senate Committee recommendation was for the Department of Health and Ageing to prepare a brief for COAG’s Standing Council on Health on existing or emerging gaps affecting the delivery of health services to rural and remote communities caused by mis-alignment between Commonwealth and state policy, including options for measures to remediate such gaps [21].

Antioch [22] recommended to the 2013 Senate Inquiry on the Aged Care Quality Agency Bill 2013 that the Agency, which is the new accreditation body, should include health economists and indigenous health experts and should collaborate with the NHMRC, GENI, Australian Commission on Safety and Quality in Health Care (ACSQHC), Cochrane Collaboration, Guidelines International Network (GIN), National Guideline Clearing House AHRQ, NICE, NHS Economic Evaluation Database (NHS EED), NHS Evidence, WHO Health Economics Network and the CDC. That Committee considered five Bills on Aged Care of which the Quality Agency was one. The Senate Committee’s Report recommended monitoring the impacts of fee scales on client welfare and providers, and new supplements for the homeless and workforce. The report addressed guidelines from the perspective of home care packages based on clinical need, accreditation of residential care services, workforce supplements and pricing [23].

Under the April 2016 COAG heads of agreement, which cover public hospital funding from 2017 to 2020, the Commonwealth will provide $2.9 b in additional funding but growth is capped at 6.5% with some funding linked to quality outcomes. The agreements will reduce payments for specified adverse events, ineffective interventions, procedures known to be harmful and readmission rates and the Independent Hospitals Pricing Authority (IHPA) is working with the ACSQHC and COAG to develop implementation details [24], Antioch [24] recommended the risk adjustment of all comparative hospital data to capture, inter alia, the impact of state-wide referral services on case-mix severity and emphasised that reductions in funding can unfortunately further exacerbate adverse quality outcomes for some conditions such as certain infections. The Committee concluded that ‘the gap in health and education funding remains. The Government remains committed to policies that deprive these areas of the much needed funds’ [25].

In response to the Senate Select Committee on Health Inquiry on Health Policy, Administration and Expenditure in 2016, Antioch [26] recommended that, in conjunction with the COAG 2016 Health Agreements and associated ABF linking risk adjusted quality outcomes to funding, COAG should facilitate dissemination of NHMRC’s methods for using the costs and benefits evidence in CPGs Australia-wide and the revised methods once finalised [25]. The Inquiry recommended that the Government should determine that the implementation mechanisms for ABF should not be dismantled and a body similar to the National Health and Hospitals Reform Commission should be reconstituted [27]. We now turn to the NHMRC review of CPG development processes in Australia.

#### NHMRC review of Australian clinical practice guideline development and approval methodologies

NHMRC is reviewing its CPG development and implementation methods, including the use of economic evidence and requested advice from GENI on use of the costs and benefits and socio-economic evidence. The advice was provided to the NHMRC in September 2013 and was based on the issues cited herein to 2013. More recent issues covered can further facilitate NHMRC’s review which is still underway. The NHMRC [28] ‘Procedures and Requirements for Meeting the 2011 NHMRC Standard for Clinical Practice Guidelines document provides direction for CPG developers who must initially register their guidelines in the NHMRC Development Register and include evidence about needs analysis, disease burden and the clinical problem to be addressed. NHMRC then notifies the Australian Health Ministers Advisory Council if CPGs have been accepted for consideration. The CPG drafts are assessed using the AGREE II tool. The CPG drafts are forwarded to the Therapeutics Goods Administration (TGA), Pharmaceutical Benefits Advisory Committee (PBAC), Medical Services Advisory Committee (MSAC) and Health Departments for comment. The NHMRC’s current review of its CPG processes and standards aims to improve development and implementation of CPGs, public health guidelines, systematic reviews and decision making. This review involves NHMRC Principal Committees, NHMRC Synthesis and Translation of Research Evidence Advisory Group 2014-16 with Working Groups formed from NHMRC’s Research Translation Group (RTG) Advisors List. The current NHMRC standards [28] handbooks on CPGs [5–9, 29, 30] and methodological guidance by AHRQ and NICE will be considered. The NHMRC [28] Standards refer to the publication ‘NHMRC additional levels of evidence and grades of recommendations for developers of guidelines’ (NHMRC 2009) [29]. However, NHMRC [29] does not address the economic evidence. Rather, economic evidence is only addressed in ‘How to compare the costs and benefits: evaluation of the economic evidence [9]. The NHMRC’s [28] standards state that the NHMRC desires input on CEA of interventions to be included in guidelines. Hence, economic issues are important. NHMRC Standard C. 3.4 states the evidence review should involve search strategies for cost effectiveness, and resource implications of practice. NHMRC Standard D.9.2 indicates that guideline recommendations should consider resource implications and cost effectiveness of recommended practice compared to current practice. NHMRC Standard G.4 indicates that resources for guideline implementation should be considered. The NHMRC [9] handbook on how to evaluate the economic evidence uses Drummond et al. “10 point” check list [11] and assesses whether there is a well-defined question, a comprehensive description of alternatives is provided, effectiveness is established. All relevant cost and consequences are included along with appropriate measurement. It also assesses credible valuation, differential timing, incremental costs and consequences, and whether allowance is made for uncertainty and appropriate interpretation of results [9]. The matrix in Table 1 is used to make decisions about the new technology given the strength of evidence for outcomes and costs [9]. The methods for the economic evaluation of health care programmes and treatments have also been discussed in the requirements of other decision-making bodies, such as the National Institute of Health and Care Excellence (NICE) in the UK, which uses the NICE Reference Case, considered in more detail later. The reporting of economic evaluations of health interventions is challenging because substantial information must be provided to enable the scrutiny of findings. Despite a growth in published reports in recent years, reporting guidelines have not being widely adopted. There has been a need to consolidate and update existing guidelines in a user-friendly manner. Checklists can assist peer reviews, authors, and editors, to use consistent guidelines to improve reporting. The Consolidated Health Economic Evaluation Reporting Standards (CHEERS statement consolidates and updates previous health economic evaluation guidelines into one current, useful reporting guidance.

**Table 1:** Assessing CEA evidence using shadow prices in Australia: NHMRC [9]

Ranking of evidence on costs	Ranking of evidence on effects	
	High	Low
Strong	Recommend if:	Recommend if:
	<$70,000 per life-year	<$30,000 per life-year
	Do not recommend if	Do not recommend if
	>$100,000 per life-year	>$70,000 per life-year
Weak	Recommend if:	Recommend if:
	<$30,000 per life-year	<$30,000 per life-year
	Do not recommend if	Do not recommend if
	>$70,000 per life-year	>$30,000 per life-year

The ISPOR 2013 CHEERS 24 item check list, discussed more fully in the next section, can be used by the NHMRC, in conjunction with Drummond et al’s 10 point check list, when assessing the economic evidence. A questionnaire by Antioch, Jennings, Botti et al. [10] published in the *European Journal of Health Economics,* that scores Drummond et al. [11] 10 point check list, has been used in Australia since 2000 to grade the cost effectiveness evidence using NHMRC methods and CEA thresholds for making recommendations about changes to clinical guidelines and for implementing evidence in three Victorian Local Hospital Networks. Since 2013, that questionnaire [10] has been used in Australia in conjunction with CHEERS assessments which enables an in-depth analysis of issues covered in the Drummond’s 10-point checklist. This work on grading the CEA evidence in systematic reviews to evaluate CPGs for diseases such as diabetes type 2 and cystic fibrosis involves senior members of public and private hospitals, national associations, universities, and GENI’s senior management team.

We outline below international findings that can also guide NHMRC’s review of CEA thresholds for CPG development and approval. CEA studies should identify the health benefits offered by an intervention; the additional costs imposed on a limited healthcare budget; and the opportunity costs (i.e. health benefits forgone) from commitment of resources to an intervention. An intervention can be considered cost-effective if its benefits outweigh the opportunity costs of health benefits forgone. The opportunity costs in terms of forgone health benefits are reflected in most CEAs by using a cost-effectiveness threshold (CET) [33].

When the maximum acceptable trade-off between costs and effectiveness is known, CEA can inform whether a program providing a trade-off between its costs and its effectiveness should be implemented. By using the maximum WTP per Quality-Adjusted Life Year (QALY), one can establish a decision rule for CEA. This view emanates from welfare economics, which states that welfare is maximized if goods are exchanged when there is a maximum WTP that exceeds the opportunity cost of the good [31]. The NHMRC defines its CEA threshold or ‘shadow prices’ as is the predetermined maximum WTP for health gains [9].

CEA used by NICE is an assessment of whether the health expected to be gained from using a new medical technology exceeds the health likely to be forgone as other NHS activities are displaced to accommodate the additional costs of the new technology. The CEA threshold therefore represents an estimate of the health forgone as services are displaced [32].

The challenge for decision makers is to determine and use CETs that reflect supply-side constraints. However, there are few empirically estimated supply-side CETs. One exception is Claxton et al. [33] study that estimated the marginal productivity of the UK National NHS and produced a best estimate of the supply-side CET of £12,936, about half of UK GDP pc. [32].

Claxton et al. [33] estimated the effects of changes in NHS expenditure on the health of all NHS patients and found that NICE’s CEA threshold used to assess new drugs is too high. The approval of new drugs is therefore causing more harm than good to NHS patents overall given the NHS is paying too much for new drugs. NICE has applied £30,000 per QALY threshold to determine whether health benefits of a new drug are greater than the health likely to be lost because the additional resources required are not available to offer effective treatments to other NHS patients. Claxton et al. [33] found that the threshold is too high as their best empirical estimates, based on a number of assumptions, was £12,936 (rounded £13,000) of NHS resources adds one QALY to the lives of NHS patients. Greater harm is therefore being done to other NHS patients when NICE approves costlier drugs. The approval of a new drug that costs the NHS an additional £10 m per annum would offer benefits of 333 QALYs. It would also lead to the loss of 773 QALYs for other NHS patients with increased mortality in circulatory, cancer, gastro-intestinal or respiratory diseases and reduced quality of life in mental health and neurological diseases. This represents a net loss of 440 QALY for every £10 m of additional NHS costs. Further, devoting £280 m to the Cancer Drugs Fund in 2014/15 has been associated with a loss of 21,645 QALYs for other NHS patients [33, 34].

NHMRC [9] includes additional criteria which allow higher prices for CEA thresholds for criteria such as: quality of life for patient and/or family; survival improvement; functional status; condition is severe, rare, preventable, or leads to permanent effects in youth; no other health care options available and intervention prevents adverse flow-on effects into other sectors with equity implications. Health care options might require further consideration with regard to these social values if they fall in the range $70,000–$100,000 per life-year saved and rank highly for evidence on costs and effects, or if they are in the range $30,000–$70,000 per life-year saved and rank highly on one but not the other [9].

Other developments about CEA thresholds in Australia are important to consider. Australia has public insurance coverage for drugs through the Pharmaceutical Benefits Scheme (PBS). Harris et al. [35] found that the Pharmaceutical Benefits Advisory Council (PBAC) decided drug approval coverage from 1994 to 2004 without reference to a public CEA threshold for the cost per LYG or QALY. WTP and decisions were based on the clinical significance, CEA results, cost to government and disease severity. Clinical significance increased the probability of recommending coverage by 0.21 and a drug for a ‘life threatening’ condition increased that probability by 0.38. Increases of $A10,000 from an incremental cost per QALY of $A46,400, reduced the probability of listing by 0.06 [32]. Harris et al. [36] analysed 1993–2009 PBAC funding decisions. They found that an A$10,000 increase in cost per QALY reduces average probability of funding from 37 to 33%. For life threatening conditions or where the drug has no active comparator, odds of positive recommendation are 3.18 and 2.14 greater. If both conditions are met, the odds are increased by 4.41 times [36].

Shiroiwa, Sung and Fukuda et al. [36] note that although CEA thresholds for medical interventions are assumed to be $50,000–$100,000 in the US and 20,000–30,000 UK pounds in the UK such values are unjustified, given lack of evidence. They measured WTP for one additional QALY gained to determine the threshold of the incremental cost-effectiveness ratio. They compared WTP for the additional year of survival in a perfect status of health in Japan, the Republic of Korea (ROK), Taiwan, Australia, the UK, and USA. A double-bound dichotomous choice was used, and analysis by the nonparametric Turnbull method. WTP values were JPY 5 million (Japan), KWN 68 million (ROK), NT$2.1 million (Taiwan), 23,000 UK pounds (UK), AU$64,000 (Australia), and US$62,000 (US). The discount rates of outcome were estimated at 6.8% (Japan), 3.7% (ROK), 1.6% (Taiwan), 2.8% (UK), 1.9% (Australia), and 3.2% (US). They recommended a new classification of cost-effectiveness plane and methodology for decision making [37].

Importantly, Drummond et al. [38] note that NICE has developed supplementary guidance for ‘end of life’ therapies in the circumstance where the therapy is for a small patient population with life expectancy of less than 24 months and where the therapy adds three months or more to life expectancy. In this scenario, QALYs gained should assume full quality-of-life in the added months. In addition, the Committee can consider that the QALYs gained should be weighted sufficiently high for the therapy to be approved, given NICE’s current threshold. These findings along with Harris et al. [35, 36], Shiroiwa et al. [37] could inform NHMRC’s consideration of revised thresholds for CEA in its review of CPG processes and in other countries.

We now consider the CHEERS guidelines, which consolidate previous health economic evaluation guidelines. Requirements of other decision making bodies such as NICE, which uses the NICE Reference Case are also considered and may be instructive for deliberations of other CPG Agencies, including the NHMRC.

### Consolidated guidelines for the reporting of economic evaluations: the CHEERS task force—reporting guidelines for economic evaluation

The ‘*CHEERS Elaboration and Explanation Report of the ISPOR Health Economic Evaluation Publication Guidelines Good Reporting Practices Task Force’* facilitates the use of the CHEERS statement by providing examples and explanations for each recommendation [39].

If economic considerations are to be incorporated into clinical guidelines, the guidelines need to reflect the current knowledge on the cost-effectiveness methodology. Therefore, guideline developers need to consult the existing literature on economic evaluation of health care treatments and programmes. The methods for the economic evaluation of health care programmes and treatments have been discussed in the academic literature [11] and in the requirements of decision-making bodies, such as NICE in the UK which uses the NICE Reference Case [40]. The NICE Reference Case requires submission for product review to conform to the following standards: a decision problem defined by NICE scope; comparator therapies listed in NICE scope; inclusion of all direct health effects for patients and caregivers; costs incurred by NHS and Personal Social Services (PSS), cost-utility analysis with full incremental analysis; time horizon long enough to reflect all important differences in costs and outcomes between technologies being compared; claims for health effects based on systematic review; health effects expressed as QALYs with EQ-5D the preferred instrument; data for health related quality of life measurement reported by patients and/or caregivers; preference data for valuation of changes in health related quality of life obtained from a representative sample of UK population; QALYs all have equal equity weight; resource use and costs valued using prices relevant to NHS and PSS; costs and benefits should be discounted at same annual rate (3.5%) (NICE [40], Langley [42]).

Application of these standards for chronic disease interventions involves modelling the natural disease progression and the impact of competing interventions over the patient’s lifetime or similar long-term time horizon. Disease progression stages can involve a Markov process to track a hypothetical cohort of patients through disease stages. Each health state is defined via associated utilities and costs, with results expressed as cost per QALY [42]. The Markov process often involves two methods of evaluation. *Cohort simulation* tracks a hypothetical cohort of patients simultaneously through the model. *Monte Carl simulation* randomly selects a patient from the cohort and each patient transits through the model one at a time [43]. By application of a WTP threshold cost per QALY, products are judged acceptable, rejected or accepted after agreement on a the actual price set [41].

Although various aspects of the methods of economic evaluation are a continuing source of debate, there is much more agreement about the need for reporting standards for studies. That is, although different studies may use different methods, all researchers should have an obligation to report their studies in a transparent fashion. This will help guideline developers and other users of economic evaluations to benefit the most from the existing literature. The most recent attempt to specify reporting guidelines for economic evaluations is the CHEERS initiative [39].

The CHEERS Standards were developed by the Good Research Practices Task Force established by the International Society for Pharmacoeconomics and Outcomes Research (ISPOR). The Task Force comprised specialists in the economic evaluation of health care programmes and the editors of journals publishing cost-effectiveness studies of health care interventions. The reporting guidelines were developed through a process consistent with that of the CONSORT initiative for developing guidelines for the reporting of clinical trials [41].

The CHEERS guidelines shown in Additional file 1: Appendix S1 assess studies with regard to conflict of interest and funding sources in addition to traditional components of published health economic evaluations that involve the title, abstract, introduction, methods and discussion. Economic evaluation methods are assessed with regard to the target population and sub-groups, setting and location, study perspective, comparators, time horizon, discount rate, choice of health outcomes, measurement of effectiveness and evaluation of preference based outcomes, estimates of resource and costs, currency, price date, conversions, choice of model, assumptions and analytic methods. Results are assessed in terms of study parameters, incremental costs and outcomes and characterising uncertainty and heterogeneity. Research findings, limitations, generalizability and current knowledge are assessed in the study’s discussion [41].

One point to note is that, on occasions, the guidelines differ for economic evaluations conducted alongside a single clinical study (e.g. a clinical trial) and those conducted as part of a decision analytic model, involving the synthesis of data from a number of sources. Several issues arose during the development of the CHEERS guidelines. The main issue related to whether reporting guidelines should merely require the authors of a study to describe their methods, or whether authors should describe *and explain* why they had adopted a particular approach. A good example of this is the selection of comparators. The CHEERS group felt that, in addition to describing the comparators included in their study, authors should also give their rationale for the selection e.g. One rationale would be to include the current standard of care in the jurisdiction where the study was conducted [39]. The CHEERS checklist enables detailed analysis of the items in Drummond’s 10 point checklist thereby facilitating comprehensive scoring of Drummond’s checklist using the EJHE questionnaire [10] to grade the evidence.

### Structuring complex evidence and values using multi-criteria decision analysis (MCDA)

During the 1970s and 1980s there was debate in the economics and ethics literature about relevant criteria for making resource allocation decisions in health care. At that time the focus was on clinical and cost-effectiveness. During the subsequent two decades, health technology assessment bodies emerged. There was growing recognition that other criteria are important, relating to equity, acceptability, burden and sustainability. More recently during the 2010s there has been growing interest in decision analytic methods for considering multiple criteria, driven primarily by NICE in the UK and shifts to Value-Based Pricing. MCDA is a methodology designed to help decision-makers when making complex choices—first developed in the 1960s and 1970s [44]. Health care decisions are complex and often involve trade-offs between conflicting and multiple objectives. Decision making involving either committees or individuals can involve difficulty in systematically evaluating all the relevant information. The MCDA confronts trade-offs between alternatives under consideration and each decision maker prioritizes the most important.

Where a group is involved, the priorities of decision makers can conflict, rendering the decision-making process very complex. Reliance on informal processes during decision making can result in suboptimal decisions. A formal process such as MCDA is therefore required to evaluate alternatives and priorities to avoid inconsistency, variability, or a lack of predictability on the importance of specific factors or criterions in the decision. MCDA provides clarity on which criteria are relevant, their relative importance and how the information can be used in a framework for assessing available alternatives. It is an extension of decision theory that covers any decision with multiple objectives (See Thokala et al. [45] for a review).

Whilst MCDA is used extensively in other sectors, recently health care applications have increased. During 2014, the International Society for Pharmacoeconomics and Outcomes Research (ISPOR) established an MCDA Emerging Good Practices Task Force to establish a common MCDA approach in health care and develop associated good practice guidelines. MCDA is applied in various types of decision making in health care, including benefit risk analysis, health technology assessment, resource allocation, portfolio decision analysis, shared patient clinician decision making and prioritizing patients’ access to services. It allows for the inclusion of preferences and social values [45].

### MCDA approaches

MCDA approaches can be classified into value measurement models, outranking models, and reference-level models [46].

- *Value measurement models* develop and compare numerical scores to determine the extent to which one decision alternative is preferred over another. They often involve additive models—“weighted-sum” models, or “additive multi-attribute value models”. These multiply a numerical score for each alternative on a specific criterion by the relative weight for the criterion and then sum those weighted scores to determine a “total score” for each alternative.
- *Reference*-*level modelling* searches for the alternative that is closest to attaining pre-defined minimum levels of performance on each criterion, using linear programming techniques and aspiration or goal methods.
- *Outranking methods* make pair-wise comparisons of alternatives on each criterion which are combined to measure support for each alternative being judged the top-ranked alternative. Outranking algorithms include the Elimination and Choice Expressing Reality (ELECTRE) methods, Preference Ranking Organization Method for Enrichment of Evaluations (PROMETHEE), and geometrical analysis for interactive aid (GAIA) (See Thokala et al. for a review) [45].

Value measurement approaches are the most widely used approach in health care. However, determining which MCDA model is most appropriate depends on the analysis and decision maker preferences [46].

Decision-makers in the health sector face a global challenge: how do we develop robust, evidence-based, scientific methods for priority setting? This challenge is often most evident in decisions concerning the coverage/reimbursement of new drugs and technologies. Peacock, Mitton and Cromwell et al. [44] indicated that a range of different criteria may be relevant for such decisions, including the effectiveness, cost-effectiveness and budget impact of the new drug; the incidence/prevalence and severity of the disease; the population group affected; the availability of alternative drugs/technologies; and, the quality of the available evidence [44].

When comparing new versus existing drugs/technologies these criteria will often need to be traded-off against one another. For example, a new drug may be more effective, but it may be costlier and targeted towards a smaller sub-population. MCDA can assist decision-makers make trade-offs when resources are constrained. When such trade-offs exist, MCDA offers a structured approach to evaluating and identifying a preferred option by scoring and weighting the various attributes and deriving an aggregate ‘value’. This is in contrast with more traditional committee based approaches, where trade-offs either not discussed or are explored in a more qualitative manner.

Some key methodological issues and challenges identified by Peacock et al. [44]. There are two main stages associated with MDCA, i.e. problem structuring and model building. Problem structuring involves generating a set of alternatives and a set of criteria against which the alternatives are evaluated and compared. Model building involves constructing some form of model which represents decision-makers’ objectives and their value judgements. A key consideration is the methods used to describe decision-makers preferences and ‘importance’ weights for decision-making criteria and the type of aggregation model used to combine criteria scores.

A simple model is:

1 $$WBS_{j} = \sum\limits_{i = 1}^{n} {w_{i} s_{ij} }$$

where i = 1,…, n criteria; w_i_ = criteria weights; j represents alternatives; s_ij_ = scores for alternatives for different criteria; WBS = Weighted Benefit Score.

Priority setting can be conceptualized as a continuous quality-improvement reiterative process involving the following sequential steps: define the aim and scope; form an advisory panel of stakeholders; establish a program budget, develop the decision criteria; identify and rank options; make decisions and specify the rationale; undertake a decision review process and finally, evaluate and improve. The economic evidence and MCDA are applied at the decision criteria development stage through to decision making and rationale specification stage. A review of the literature identified 52 different criteria listed in 14 studies, with the most commonly occurring criteria, in descending order, including accessibility, reducing inequalities, effectiveness, alignment with strategic plan and policies, value for money, affordability and integration with other programs. Peacock et al. [44] compared criteria used in Australia and Canada for cancer control and also for HTA agencies in Australia and the UK. These criteria are in Table 2.

**Table 2:** Criteria developed by Cancer Control and HTA Agencies

Cancer control initiatives	National HTA agencies
National cancer control initiative (Australia) Health gain-disability adjusted life years (DALYs) (Vertical) equity-reducing inequalities in mortality rates in vulnerable populations Size of health burden-disease burden in DALYs and cost to health system Acceptability and feasibility	NICE (England and Wales) Clinical effectiveness Cost-effectiveness Feasibility and impact Equity and equality Acceptability and appropriateness
British Columbia cancer control decision-makers (Canada) Health gain-quality adjusted life years (QALYs) Resource impact-budget constraints Resource impact- resource constraints Availability/advisability of alternatives	Pharmaceutical benefits advisory committee (Australia) Gap in current coverage Cost-effectiveness Effectiveness and safety Community need

A review of the literature concerning criteria weights in MCDA methods found that four studies did not report weights and seven studies used allocation of points (direct rating). Two studies used a combination of ratio estimation and direct rating. One study used indifference methods (DCEs). Previous studies have also used swing weights (hybrid of indifference method). No studies have used gambles—all choices riskless [44].

With regard to MCDA methods for aggregation rules, Peacock et al. [44] found that where the aggregation rule was presented almost all (i.e. nine) applications used an additive functional form. Three did not state the functional form used. One used an exponential function and another used a variant of the multiplicative function. Peacock et al. found that the choice of functional form was rarely justified [44].

MCDA is useful from several perspectives. The primary aim of MCDA is to develop models of decision-maker objectives and their value trade-offs so that alternatives under consideration can be compared with each other in a consistent and transparent manner. The process is often more important than the numbers. It involves value focussed thinking and values clarification. MCDA practice suggests preferences are constructed as part of the decision-making process, not endowed. It is consistent with deliberative-analytic methods.

There are several issues that have arisen involving international consistencies and controversies in the decision criteria used in priority setting, the methods used to elicit criteria weights, and the complexity and transparency of the decision-making process. These fall into four broad methodological challenges. Firstly, how do we decide which stakeholder’s criteria will count—society, governments, private sector, patients, families, health providers, or other decision-makers? Second, which methods should be used to elicit and describe decision-makers preferences, including the relationship between objectives and criteria. Further, the methods used to elicit importance weights for decision-making criteria can be controversial along with the type of aggregation model used to combine criteria scores.

Is there any common ground? Economics has often focussed on prescriptive behavioural rules, based on utility maximisation and game theory. On the other hand, psychology has sought to explain actual individual behaviour, and why it can deviate from prescriptive rules. Interestingly, decision analysis, including MCDA, endeavours to combine elements of both approaches through both ‘prescription’ with ‘practicality’. These approaches all share a common heritage from von Neumann and Morgenstern [44].

ISPOR’s 2016s Task Force report provides good-practice guidance on the implementation of MCDA for health care decisions. It incorporates a checklist to support the design, implementation and review of an MCDA; guidance to support checklist implementation; the sequencing of implemented steps and describes the incorporation of budget constraints into an MCDA. It also covers the skills and resources, including software, required to implement MCDA; and canvasses future research directions. ISPOR’s MCDA Good Practice Guidelines checklist is shown in Table 3 below. ISPOR also provides general guidance on the validation process in each step and on how to implement the other recommendations in the checklist [47].

**Table 3:** ISPOR MCDA good practice guidelines checklist (Source: Marsh et al. [47])

Defining the decision problem	Develop a clear description of the decision problem
	Validate and report the decision problem
Selecting and structuring criteria	Report and justify the methods used to identify criteria
	Report and justify the criteria definitions
	Validate and report the criteria and the value tree
Measure performance	Report and justify the sources used to measure performance
	Validate and report the performance matrix
Scoring alternatives	Report and justify the methods used for scoring
	Validate and report scores
Weighting criteria	Report and justify the methods used for weighting
	Validate and report weights
Calculating aggregate scores	Report and justify the aggregation function used
	Validate and report results of the aggregation
Dealing with uncertainty	Report sources of uncertainty
	Report and justify the uncertainty analysis
Reporting and examining of findings	Report the MCDA method and findings
	Examine the MCDA findings

Several areas for further research have been identified by Marsh et al. [47] to address the challenges associated with the technique, including the level of precision required of an MCDA; the cognitive challenges facing different types of stakeholders and the support that can overcome these challenges; decision makers’ preferences for the theoretical foundations of MCDA methods; which value functions best describe stakeholders preferences; and the best methods for incorporating uncertainty and budget constraints into an MCDA. Further, constructing a cost-benefit ratio using MCDA outputs faces several challenges including the different scales that are used to measure benefits and costs [47].

### Inclusion of economic evidence in systematic reviews: recommendations after reviews on health policy impact and best practices

Systematic reviews are important in improving understanding of comparative effectiveness and relative value of medical interventions in health policy decisions and health guidelines [48]. A study by the Hopkins AHRQ project team addressed the appropriateness of incorporating economic data into systematic reviews of medical interventions in America. First, it examined if and how economic evidence impacts on health policy, including clinical guidelines, in the USA. It examined strengths and weaknesses of existing checklists used to assess best practices in economic evaluations. Finally, it reviewed studies addressing the use of economic outcomes in policy and decision making. The work is reported in publications by Niessen et al. [49], Walker et al. [50] and Frick et al. [51].

The review on *economic evidence impacts on policy including clinical guidelines*, used original studies applying quantitative or qualitative methods providing empirical data, in any country. The team excluded opinion or experienced-based articles without newly generated data. They defined ‘best-practice’ checklists as any original listing of recommendations that the authors used in economic evaluation. MEDLINE, EconLit, Cumulative Index to Nursing and Allied Health Literature (CINAHL), Embase^®^, and ISI Web of ScienceSM searched 1991 to January 2012. The literature was searched for articles on economic evaluations, outcomes, and guidelines for the decision maker. Paired reviewers independently determined whether articles met eligibility criteria and then extracted data.

Reviewers assessed the quality of each reporting study on policy impact using the standard grading recommendations (GRADE). Of 19,127 titles initially screened, 43 studies on policy impact were included, with all but five published since 2000. The most frequently studied countries were the United Kingdom (15), and Australia, Canada, and the United States (5 each). Most studies (27 studies) considered national-level policy and examined the key health actors involved. Important topics were reimbursement and health package decisions, and priority setting in programming. Thirty studies found evidence that use of economic evidence had a substantial impact on health care policy making, 27 of which emphasized at least one other criterion, such as equity considerations, usually ill-defined (14 studies), clinical effectiveness, budget impact, ethical reasons, and advocacy arguments. The thirty studies confirmed the acceptance of economic evidence as having an impact on either general polic*y* or specific decisions, such as reimbursement decisions. Of 37 observational studies on policy impact, 11 (30%) received a favourable rating on more than three of the 8 items on the GRADE quality checklist. Five studies were graded of intermediate quality evidence [49].

The study concerning best practices for conducting economic evaluations in health care was a systematic review of quality assessment tools [50]. The objective was to describe strengths and weaknesses of checklists for conducting and reporting on economic evaluations in health care. The authors defined checklists as the original listings of specific items recommended in the conduct or reporting of an economic evaluation.

The study involved a review of the criteria for judging that an economic evaluation is of sufficiently high quality to be useful. It also considered the importance of different aspects of the evaluation; and the extent to which high quality on one aspect of an evaluation can compensate for lower quality items. The methods involved a systematic search until January 2012 for articles and guidelines for decision makers. In MEDLINE, EconLit, CINAHL, Embase, and ISI Web of Science. They found that ten peer-reviewed journal articles reported an original checklist. The first in 1992 and last in 2011 (EVEREST and CHEERS in 2013). The number of items ranged from 11 to 57. Perspective was a criterion in all 10. The ten checklists reviewed included those by Adams [52], Gerard [53], Sacristan [54], Clemens [55]. The U.S. Panel, the British Medical Journal (BMJ) Checklist, The Paediatrics’ Quality Appraisal Questionnaire (PQAQ). The Quality of Health Economic Studies (QHES) list. The Consensus on Health Economic Criteria (CHEC) list and a checklist to frame health technology assessments for resource allocation decisions [52–62].

Eleven criteria were in 7–9 lists: including target population, alternatives, study question, design, measurement, valuation, outcome identification, outcome measurement, time adjustment, sensitivity and uncertainties, presentation of results, generalizability, and incremental analysis. Four had evidence of excellent test–retest reliability: none had evidence of excellent inter-rater reliability in two or more studies. Three had evidence of excellent criterion validity, comparing checklists or expert ratings. They concluded that several well-developed checklists exist for investigators, reviewers, and journal editors to use in efforts to ensure more informative and transparent evidence [50].

They also analysed the usefulness of economic evaluation data in systematic reviews of evidence [51]. This addressed the question: How useful is incorporating economic evaluation data into systematic reviews of medical interventions? The method used was a consensus process with outside experts to develop a conceptual framework.

They found that all stakeholders, including insurers are interested in economic data and the perspectives of patients, providers, and manufacturers. Patients and providers determine the demand for care and manufacturers determine the supply. Decisions are to be based on incremental cost, magnitude of the incremental effect, and the probability that economic evidence will change a decision. There is a high priority for evidence if there is a small effect at a high level of expenditure with a high probability of influencing a decision.

Economic data are of interest at many policy levels including approval and monitoring of services, formulary inclusion, insurance coverage, reimbursement rate, preferred practice guideline, technology adoption or non-adoption, or clinical management. They may include information on cost-effectiveness, productivity changes related to disease, price data, responses to price changes. The following areas were of greatest interest: demand for care: time, distance, innovation price, price sensitivity, substitutes and complements, WTP, absenteeism and presentism. Supply of care was of interest including economy of scale, efficiency changes and return on investment. The equity and efficiency measures of particular interest were cost of illness studies; disease burden, Quality of life, budget impact, CEA ratio, net benefits and disparities. Existing economic data may be sufficient. Occasionally, it may be necessary to perform new evaluations in case of inadequate data [51].

## Conclusion

There is some evidence that Drummond’s 10-point checklist [11], when used in conjunction with Antioch et al.’s [10] questionnaire that scores Drummond’s checklist, has resulted in cost-effective health care. These were used to grade economic evaluations, while integrating economic evidence into CPGs by the Australian Local Hospital Networks that use both NHMRC and international methodologies. That checklist and questionnaire can be used, in conjunction with the CHEERS 24 item checklist by health services, governments, NHMRC, other CPG organisations, and the ACSQHC for grading the economic evidence. Whilst the ten checklists reviewed by AHRQ are very useful, their limitations of inter-rater reliability can be addressed by including more than one assessor to reach a consensus on assessments. Especially when impacting on treatment decisions. Further research could assess the impact of such checklists on the cost effectiveness of health services where they have been used to assess evidence for CPGs and treatment decisions. Antioch et al. [10] questionnaire involves consensus by two assessors, given the impacts on treatment decisions. The issues discussed concerning CEA thresholds, decision rules, opportunity costs, WTP and end of life therapies are also instructive world-wide. Priority setting is important and where trade-off decisions between criteria are required and MCDA is useful for Evidence Based Medicine and Evidence Based Planning. The Hopkins review identifies key priority areas for studies including the demand for care issues which can investigate time, distance, innovation price, price sensitivity, substitutes and complements, WTP, absenteeism and presentism. Supply of care issues can explore economies of scale, efficiency changes, and return-on-investments, equity and efficiency measures can include cost of illness studies, disease burden, quality of life, budget impact, CEA ratios, net benefits and disparities.

COAG, AHMAC, IHPA, ACSQHC and the Australian Department of Health could expedite the Australia-wide dissemination of NHMRC’s methods on using the costs and benefits in CPGs and the subsequent revised methods. This may assist the health industry prepare for implementation of COAG’s 2016 Heads of Agreements on Public Hospital Funding which reduces ABF for specified adverse events, ineffective or harmful treatments and readmissions. IHPA, ACSQHC and COAG can risk adjust ABF and quality outcome measurement, through analysis of complexity markers for state-wide referral services such as transplantation, major trauma and cystic fibrosis to enable equity.

## Additional file

**Additional file 1: Appendix S1.** CHEERS checklist—items to include when reporting economic of health intervention.

## Abbreviations

ABF: activity based funding
AHCA: Australian Health Care Agreements
AHRQ: Agency of Health Research and Quality
CAHRD: Centre for Applied Health Research and Delivery
CBA: cost benefit analysis
CDC: Centre for Disease Control and Prevention
CEA: cost-effectiveness analysis
CHEC: Consensus on Health Economic Criteria List
CHEERS: Consolidated Health Economic Evaluation Reporting Standards
CINAHL: Cumulative Index to Nursing and Allied Health Literature (CINAHL)
COAG: Council of Australian Governments
COHERE: Center for Health Economic Research
CPG: Clinical Practice Guidelines
DALY: disability adjusted life year
DCG-HCC: diagnostic cost group-hierarchical condition categories
EBM: evidence-based medicine
ELEC|TR|E: elimination and choice expressing reality
GAIA: geometrical analysis for interactive aid
GENI: Guidelines and Economists Network International
GIN: Guidelines International Network
GRADE: Grading of Recommendations Assessment, Development and Evaluation
IHEA: International Health Economics Association
ISPOR: International Society for Pharmacoeconomics and Outcomes Research
LHN: local hospital networks
MCDA: multi-criteria decision analysis
NHMRC: National Health and Medical Research Council’s
NHS: National Health Services
NHSEED: National Health Services Economic Evaluations Database
NICE: National Institute for Health and Care Excellence
PQAQ: Paediatrics Quality Appraisal Questionnaire (PQAQ)
PROMETHEE: Preference Ranking Organization Method for Enrichment of Evaluations
QALY: quality adjusted life year
RTG: Research Translation Groups
WTP: willingness-to-pay

## Footnotes

1
http://geni-econ.org/.

2 This includes the Agency for Clinical Innovation (ACI) and Centre for Health Care Redesign in NSW and the Centre for Health Care Improvement in Queensland. In Victoria the Commission for Hospital Improvement and the Health Innovation and Reform Council were created. More recently the Victorian Commission was disbanded and replaced by Better Care Victoria.

## References

[1] Council of Australian Governments (COAG). National Health Reform Agreement between the Commonwealth of Australian and State and Territory Governments. 2011. http://www.federalfinancialrelations.gov.au/content/npa/health_reform/national-agreement.pdf. Accessed 15 May 2015.

[2] Antioch KM. Integrating Economic and Clinical Evidence, Guidelines and Equity into National Regulation and Financing: Reforms for the Australian Health Care Agreements (AHCA): 2009 and Beyond. Paper to Council of Australian Government (COAG), State and Federal stakeholders. Published by National Health and Hospitals Reform Commission (NHHRC) and Department of Health and Ageing (DOHA) with permission of Department of Prime Minister and Cabinet (DPMC). 2008. http://www.health.gov.au/internet/nhhrc/publishing.nsf/Content/297-interim/%24FILE/298%20-%20Submission%20-%20%20Dr%20Kathryn%20Antioch.pdf. Accessed 15 May 2015.

[3] Antioch KM. Intergovernmental agreements: update on reforms on risk adjustment of health funding and evidence based medicine (EBM) Implementation. Paper to COAG; State and Federal stakeholders. (Published by NHHRC and DOHA with permission of DPMC). 2009. http://www.health.gov.au/internet/nhhrc/publishing.nsf/Content/297-interim/%24FILE/297%20-%20Submission%20-%20%20Dr%20Kathryn%20Antioch.pdf. Accessed 15 May 2015.

[4] Antioch KM. COAG: April 2010 update on reforms on activity based funding, risk adjustment and evidence based medicine (EBM) implementation’. Paper to COAG; State and Federal stakehoders for April 2010 COAG meeting, Canberra. (Submission published by Australian Senate Finance and Public Administration References Committee). 2010a. See Submission 20 http://www.aph.gov.au/Parliamentary_Business/Committees/Senate/Finance_and_Public_Administration/Completed%20inquiries/2008-10/coag_health_reforms/submissions. Accessed 15 May 2015.

[5] NHMRC. A guide to the development, implementation and evaluation of clinical practice guidelines. Canberra: National Health and Medical Research Council. 1999. https://www.nhmrc.gov.au/_files_nhmrc/publications/attachments/cp30.pdf. Accessed 15 May 2015.

[6] NHMRC. How to put the evidence into practice: implementation and dissemination strategies Handbook series on preparing clinical practice guidelines. Canberra National Health and Medical Research Council. 2000a. https://www.nhmrc.gov.au/_files_nhmrc/publications/attachments/cp71.pdf. Accessed 15 May 2015.

[7] NHMRC. How to review the evidence: systematic identification and review of the scientific literature. Canberra: National Health and Medical Research Council. 2000b. https://www.nhmrc.gov.au/_files_nhmrc/publications/attachments/cp65.pdf. Accessed 15 May 2015.

[8] NHMRC. How to use the evidence: assessment and application of scientific evidence. Canberra: National Health and Medical Research Council. 2000c. https://www.nhmrc.gov.au/_files_nhmrc/publications/attachments/cp69.pdf. Accessed 15 May 2015.

[9] NHMRC. How to compare the costs and benefits: evaluation of the economic evidence Handbook series on preparing clinical practice guidelines, Canberra National Health and Medical Research Council. 2001. https://www.nhmrc.gov.au/_files_nhmrc/publications/attachments/cp73.pdf. Accessed 15 May 2015.

[10] Antioch KM, Jennings G, Botti M, Chapman R and Wulfsohn V. ‘Integrating cost-effectiveness evidence into clinical practice guidelines in Australia for Acute Myocardial Infarction’. Eur J Health Eco. 2002;3:26–39. http://link.springer.com/article/10.1007/s10198-001-0088-z. Accessed 22 Sept 2016. 10.1007/s10198-001-0088-z 15609115

[11] Drummond MF, Sculpher MJ, Torrance GW, Stoddart GL, O’Brien B (2005). Methods for the economic evaluation of health care programme*s*.

[12] Antioch KM, Ellis RP, Gillett S, et al. Risk adjustment Policy Options for Casemix Funding: International Lessons in Financing Reforms. Eur J Health Eco. 2007;8: 195–212. http://people.bu.edu/ellisrp/EllisPapers/2007_AntiochEllisGillett_EJHE_RiskAdj.pdf. 10.1007/s10198-006-0020-7 17273852

[13] Independent Hospital Pricing Authority (IHPA), 2015 National Efficient Price (NEP) Determination 2015–2016. 2015. https://www.ihpa.gov.au/sites/g/files/net636/f/publications/national-efficient-price-determination-2015-16.pdf. Accessed 22 Sept 2016.

[14] Antioch KM. Invited Submission to the Senate Community Affairs Legislation Committee Inquiry into the National Health Reform Amendment (National Health Performance Authority) Bill 2011. (Published by Australian Parliament). 2011a. See Submission 14 http://www.aph.gov.au/Parliamentary_Business/Committees/Senate/Community_Affairs/Completed_inquiries/2010-13/nhpa/submissions. Accessed 15 May 2015.

[15] Antioch KM. Submission to the Senate Finance and Public Administration Legislation Committee Inquiry into the National Health Reform Amendment (Independent Hospital Pricing Authority) Bill 2011. (Published by Australian Parliament). 2011b. See Submission 14 http://www.aph.gov.au/Parliamentary_Business/Committees/Senate/Finance_and_Public_Administration/Completed%20inquiries/2010-13/indhospitalpricingauthority/submissions. Accessed 15 May 2015.

[16] Economics Legislation Senate Committee. Senate Committee Report Federal Financial Relations Amendment (National Health and Hospitals Network) Bill 2010 [provisions] pp. 9–10. 2011. http://www.aph.gov.au/Parliamentary_Business/Committees/Senate/Economics/Completed_inquiries/2010-13/healthfinance10/report/index. Accessed 15 May 2015.

[17] Antioch KM. Submission to the Senate Economics Committee Inquiry into the Federal Financial Relations Amendment (National Health and Hospitals Network) Bill 2010. (Published by Australian Parliament). 2010b. See Submission 1 http://www.aph.gov.au/Parliamentary_Business/Committees/Senate/Economics/Completed%20inquiries/2010-13/healthfinance10/submissions. Accessed 15 May 2015.

[18] Antioch KM. Submission to the Senate Community Affairs References Committee Inquiry into Palliative Care in Australia. (Published by Parliament). 2012a. See Submission 137 http://www.aph.gov.au/Parliamentary_Business/Committees/Senate/Community_Affairs/Completed_inquiries/2010-13/palliativecare/submissions. Accessed 15 May 2015.

[19] Senate Community Affairs Reference Committee. Senate Committee Report on Palliative Care in Australia. (Published by Parliament). 2012. http://www.aph.gov.au/Parliamentary_Business/Committees/Senate/Community_Affairs/Completed_inquiries/2010-13/palliativecare/report/index. Accessed 22 Sept 2016.

[20] Antioch KM. Submission to the Senate Community Affairs References Committee Inquiry into the Factors Affecting the Supply of Health Services and Medical Professionals in Rural Areas. (Published by Australian Parliament). 2012b. See submission 132. http://www.aph.gov.au/Parliamentary_Business/Committees/Senate/Community_Affairs/Completed_inquiries/2010-13/rurhlth/submissions. Accessed 15 May 2015.

[21] Senate Community Affairs References Committee. Senate Committee Report on the Inquiry into the Factors Affecting the Supply of Health Services and Medical Professionals in Rural Areas. 2012. http://www.aph.gov.au/Parliamentary_Business/Committees/Senate/Community_Affairs/Completed_inquiries/2010-13/rurhlth/report/index. Accessed 22 Sept 2016.

[22] Antioch KM. Submission to the Senate Standing Community Affairs Legislative Committee Inquiry into the Aged Care (Living Longer Living Better) Bill 2013 and four related Bills. (Published by Australian Parliament). 2013b. See Submission 107 http://www.aph.gov.au/Parliamentary_Business/Committees/Senate/Community_Affairs/Completed_inquiries/2010-13/agedcare/submissions. Accessed 15 May 2015.

[23] Senate Standing Community Affairs Legislative Report of the Committee Inquiry into the Aged Care (Living Longer Living Better) Bill 2013 and four related Bills. (Published by Australian Parliament). 2013b. http://www.aph.gov.au/Parliamentary_Business/Committees/Senate/Community_Affairs/Completed_inquiries/2010-13/agedcare/index. Accessed 22 Sept 2016.

[24] Antioch KM. Submission to Senate Finance and Public Administration Committee Inquiry on the Outcomes of the 42nd Meeting of the Council of Australian Governments (COAG) held on 1 April 2016. (Published by Parliament). 2016a. See Submission 7 2016 http://www.aph.gov.au/Parliamentary_Business/Committees/Senate/Finance_and_Public_Administration/COAG/Submissions. Accessed 22 Sept 2016.

[25] Senate Finance and Public Administration Committee Report of the Committee Inquiry on the Outcomes of the 42nd Meeting of the Council of Australian Governments (COAG) held on 1 April 2016. (Published by Parliament). 2016. http://www.aph.gov.au/Parliamentary_Business/Committees/Senate/Finance_and_Public_Administration/COAG/Report. Accessed 22 Sept 2016.

[26] Antioch KM Submission to the Select Committee on Health inquiry into health policy, administration and expenditure. 2016b. See submission 204. http://www.aph.gov.au/Parliamentary_Business/Committees/Senate/Health/Health/Submissions. Accessed 22 Sept 2016.

[27] Select Committee on Health inquiry into health policy, administration and expenditure Final report Hospital funding cuts: the perfect storm the demolition of Federal-State health relations 2014–2016. 2016. http://www.aph.gov.au/Parliamentary_Business/Committees/Senate/Health/Health/Final_Report. Accessed 22 Sept 2016.

[28] NHMRC. Procedures and requirements for meeting the 2011 NHMRC standard for clinical practice guidelines May 2011 Version 1.1 Canberra: National Health and Medical Research Council. 2011. http://www.nhmrc.gov.au/_files_nhmrc/publications/attachments/cp133_nhmrc_procedures_requirements_guidelines_v1.1_120125.pdf. Accessed 15 May 2015.

[29] NHMRC. Using socioeconomic evidence in clinical practice guidelines Canberra National Health and Medical Research Council. 2002. https://www.nhmrc.gov.au/_files_nhmrc/publications/attachments/cp89.pdf. Accessed 15 May 2015.

[30] NHMRC. NHMRC additional levels of evidence and grades of recommendations for developers of guidelines. Canberra: National Health and Medical Research Council. 2009 http://www.nhmrc.gov.au/_files_nhmrc/file/guidelines/stage_2_consultation_levels_and_grades.pdf. Accessed 15 May 2015.

[31] Polsky D. Does willingness to pay per quality-adjusted life year bring us closer to a useful decision rule for cost-effectiveness analysis? Med Decis Mak 25. 2005;6: 605–606 http://mdm.sagepub.com/content/25/6/605.full.pdf+html. Accessed 22 Sept 2016. 10.1177/0272989X05283136 16282209

[32] https://www.york.ac.uk/che/research/teehta/thresholds/. Accessed 22 Sept 2016.

[33] Claxton K, Martin S, Soares M, Rice N, Spackman E, Hinde S, Devlin N, Smith PC, Sculpher M. Methods for the estimation of the NICE cost effectiveness threshold Health Technology Assessment. Available on the NIHR Journals Library website. From the 19th of February 2015. http://www.journalslibrary.nihr.ac.uk/hta. Accessed 22 Sept 2016. 10.3310/hta19140 PMC4781395 25692211

[34] University of York Media Release Approval of new drugs by NICE is doing more harm than good. The NHS is paying too much for new drugs 19th February 2015. https://www.york.ac.uk/media/che/documents/NICE%20Threshold%20Press%20Release%20190215.pdf. Accessed 22 Sept 2016.

[35] Harris A, Hill S, Chin G, Li JJ, Walkom E. The role of value for money in public insurance coverage decisions for drugs in Australia. A retrospective analysis 1994–2004. Med Decis Mak. 2008;28(5):713–22. http://mdm.sagepub.com/content/28/5/713.short. Accessed 15 May 2015. 10.1177/0272989X08315247 18378939

[36] Harris, A Li JJ, Yong K. What Can We Expect from Value-Based Funding of Medicines? A Retrospective Study? PharmacoEconomics. 2016;34:393–402. doi: 10.1007/s40273-015-0354-z. http://link.springer.com/article/10.1007%2Fs40273-015-0354-z. Accessed 22 Sept 2016. 10.1007/s40273-015-0354-z 26610347

[37] Shiroiwa T, Sung YK, Fukuda T, Lang HC, Bae SC, Tsutani K (2010). International survey on willingness-to-pay (WTP) for one additional QALY gained: what is the threshold of cost effectiveness?. Health Econ Apr..

[38] Drummond MF, de Pouvourville G, Jones E, Haig J, Saba G, Cawston H (2014). A comparative analysis of two contrasting European approaches for rewarding the value added by drugs for cancer: England versus France. Pharmaco Econ..

[39] Husereau D, Drummond MF, Petrou S, Carswell C, Moher D, Greenberg D, Augustovski F, Briggs AH, Mauskopf J, Loder E. Consolidated Health Economic Evaluation Reporting Standards (CHEERS): explanation and elaboration: a report of the ispor health economic evaluation publication guidelines good reporting practices task force. Value Health. 2013;16:231–50. http://www.ispor.org/ValueInHealth/ShowValueInHealth.aspx?issue=3D35FDBC-D569-431D-8C27-378B8F99EC67. Accessed 15 May 2015. 10.1016/j.jval.2013.02.002 23538175

[40] National Institute for Health and Care Excellence (NICE). A guide to the methods of technology appraisal.London, July. 2013. http://www.nice.org.uk/aboutnice/howwework/devnicetech/guidetothemethodsoftechnologyappraisal.jsp. Accessed 15 May 2015. 27905712

[41] Moher D, Schulz KF, Simera I, Altman DG. Guidance for developers of Health Research Reporting Guidelines. PLoS Med. 2010;7(2):e1000217. http://journals.plos.org/plosmedicine/article?id=10.1371/journal.pmed.1000217. Accessed 15 May 2015. 10.1371/journal.pmed.1000217 PMC2821895 20169112

[42] Langley P (2016). Sunlit uplands: the genius of the NICE reference case. Innovat Pharm..

[43] Drummond M, McGuire A (2006). Economic evaluation in health care merging theory with practice.

[44] Peacock S, Mitton C, Cromwell I. Structuring complex evidence and values using multi-criteria decision analysis (MCDA) presentation in the organised session. Economic Guidelines for Clinical Guidelines 2013 International Health Economic Association (IHEA), Sydney; 2013.

[45] Thokala P, Devlin N, Marsh K, et al. Multiple criteria decision analysis for health care decision making—an introduction:report1 of the ISPOR MCDA emerging good practices task force value in health 1. 2016;9:1–1. 3 ISPOR Task Force Report. https://www.ispor.org/Multi-Criteria-Decision-Analysis-guideline.pdf. Accessed 22 Sept 2016. 10.1016/j.jval.2015.12.003 26797229

[46] Thokala P, Duenas A. Multiple criteria decision analysis for health technology assessment. Value Health 2012;15:1172–81. http://www.ispor.org/ValueInHealth/ShowValueInHealth.aspx?issue=B5983BEF-1145-4724-A446-24BFC33BE924. Accessed 22 Sept 2016. 10.1016/j.jval.2012.06.015 23244821

[47] Marsh KI, Jzerman M, Thokala P, et al. Multiple criteria decision an analysis for health care decision making—emerging good practices: report 2 of the ISPOR MCDA emerging good practices task force value in health. 2016;19:125–37. 2016 ISPOR Task Force Report. https://www.ispor.org/Multi-Criteria-Decision-Analysis-guideline-2.pdf. Accessed 22 Sept 2016. 10.1016/j.jval.2015.12.016 27021745

[48] Niessen LW (2013). Inclusion of economic evidence in systematic reviews? Recommendations after reviews on health policy impact and best practices: presentation in the organised session on “Economic Guidelines for Clinical Guidelines”.

[49] Niessen LW, Bridges J, Lau BD, Wilson RF, Sharma R, Walker DG, Frick KD, Bass EB. Assessing the impact of economic evidence on policymakers in health care—a systematic review. AHRQ Publication No. 12(13)-EHC133-EF. 2012. http://www.ncbi.nlm.nih.gov/books/NBK114636/. Accessed 15 May 2015. 23236642

[50] Walker DG, Wilson RF, Sharma R, Bridges J, Niessen L, Bass EB, Frick K. Best practices for conducting economic evaluations in health care: a systematic review of quality assessment tools. AHRQ Publication No. 12(13)-EHC132-EF. 2012. http://www.ncbi.nlm.nih.gov/pubmed/23230577. Accessed 15 May 2015. 23230577

[51] Frick K, Niessen L, Bridges J, Walker D, Wilson RF, Bass EB. Usefulness of economic evaluation data in systematic reviews of evidence. AHRQ Publication No. 2012;12(13)-EHC114-EF. http://www.ncbi.nlm.nih.gov/books/NBK114533/. Accessed 15 May 2015. 23230578

[52] Adams ME (1992). Economic analysis in randomized control trials. Med Care.

[53] Gerard K (1992). Cost-utility in practice: a policymaker’s guide to the state of the art. Health Policy.

[54] Sacristán JA, Soto J, Galende I (1993). Evaluation of pharmacoeconomic studies: utilization of a checklist. Ann Pharmacother.

[55] Clemens K, et al. Methodological and conduct principles for pharmacoeconomic research. Pharmaceutical research and manufacturers of America. Pharmacoeconomics. 1995; 8: 169–74. http://link.springer.com/article/10.2165/00019053-199508020-00008. Accessed 22 Sept 2016. 10.2165/00019053-199508020-00008 10155611

[56] Russell LB (1996). The role of cost-effectiveness analysis in health and medicine. Panel on cost-effectiveness in health and medicine. JAMA.

[57] Siegel JE (1996). Recommendations for reporting cost-effectiveness analyses. Panel on cost-effectiveness in health and medicine. JAMA.

[58] Drummond MF, Jefferson TO (1996). Guidelines for authors and peer reviewers of economic submissions to the BMJ. The BMJ economic evaluation working party. BMJ.

[59] Ungar WJ, Santos MT (2003). The pediatric quality appraisal questionnaire: an instrument for evaluation of the pediatric health economics literature. Value Health..

[60] Chiou CF, Hay JW, Wallace JF (2003). Development and validation of a grading system for the quality of cost-effectiveness studies. Med Care.

[61] Evers S, Goossens M, de Vet H (2005). Criteria list for assessment of methodological quality of economic evaluations: consensus on Health Economic Criteria. Int J Technol Assess Health Care.

[62] Grutters JP, Seferina SC, Tjan-Heijnen VC, van Kampen RJ, Goettsch WG, Joore MA (2011). Bridging trial and decision: a checklist to frame health technology assessments for resource allocation decisions. Value Health..

