# Supplementary material for: International lessons in new methods for grading and integrating cost effectiveness evidence into clinical practice guidelines
Source: Cost Eff Resour Alloc. 2017 Feb 10;15:1. doi: 10.1186/s12962-017-0063-x (PMC5303215; doi:10.1186/s12962-017-0063-x)
Supplement: Supplementary file 1 — Additional file 1: Appendix S1. CHEERS checklist—items to include when reporting economic of health intervention. [file 12962_2017_63_MOESM1_ESM.pdf]

Section/item	Item no.	Recommendation	Reported on page no./line no.
Title and abstract			
Title	1	Identify the study as an economic evaluation, or use more specific terms such as "cost-effectiveness analysis" and describe the interventions compared.	______
Abstract	2	Provide a structured summary of objectives, perspective, setting, methods (including study design and inputs), results (including base-case and uncertainty analyses), and conclusions.	______
Introduction			
Background and objectives	3	Provide an explicit statement of the broader context for the study.	______
		Present the study question and its relevance for health policy or practice decisions.	______
Methods			
Target population and subgroups	4	Describe characteristics of the base-case population and subgroups analyzed including why they were chosen.	______
Setting and location	5	State relevant aspects of the system(s) in which the decision(s) need(s) to be made.	______
Study perspective	6	Describe the perspective of the study and relate this to the costs being evaluated.	______
Comparators	7	Describe the interventions or strategies being compared and state why they were chosen.	______
Time horizon	8	State the time horizon(s) over which costs and consequences are being evaluated and say why appropriate.	______
Discount rate	9	Report the choice of discount rate(s) used for costs and outcomes and say why appropriate.	______
Choice of health outcomes	10	Describe what outcomes were used as the measure(s) of benefit in the evaluation and their relevance for the type of analysis performed.	______
Measurement of effectiveness	11a	*Single study–based estimates*: Describe fully the design features of the single effectiveness study and why the single study was a sufficient source of clinical effectiveness data.	______
	11b	*Synthesis-based estimates*: Describe fully the methods used for the identification of included studies and synthesis of clinical effectiveness data.	______
Measurement and valuation of preference-based outcomes	12	If applicable, describe the population and methods used to elicit preferences for outcomes.	______
Estimating resources and costs	13a	*Single study–based economic evaluation*: Describe approaches used to estimate resource use associated with the alternative interventions. Describe primary or secondary research methods for valuing each resource item in terms of its unit cost. Describe any adjustments made to approximate to opportunity costs.	______
	13b	*Model-based economic evaluation*: Describe approaches and data sources used to estimate resource use associated with model health states. Describe primary or secondary research methods for valuing each resource item in terms of its unit cost. Describe any adjustments made to approximate to opportunity costs.	______
Currency, price date, and conversion	14	Report the dates of the estimated resource quantities and unit costs. Describe methods for adjusting estimated unit costs to the year of reported costs if necessary. Describe methods for converting costs into a common currency base and the exchange rate.	______
Choice of model	15	Describe and give reasons for the specific type of decision-analytic model used. Providing a figure to show model structure is strongly recommended.	______
Assumptions	16	Describe all structural or other assumptions underpinning the decision-analytic model.	______
Analytic methods	17	Describe all analytic methods supporting the evaluation. This could include methods for dealing with skewed, missing, or censored data; extrapolation methods; methods for pooling data; approaches to validate or make adjustments (e.g., half-cycle corrections) to a model; and methods for handling population heterogeneity and uncertainty.	______

Section/item	Item no.	Recommendation	Reported on page no./line no.
Results			
Study parameters	18	Report the values, ranges, references, and if used, probability distributions for all parameters. Report reasons or sources for distributions used to represent uncertainty where appropriate. Providing a table to show the input values is strongly recommended.	____________
Incremental costs and outcomes	19	For each intervention, report mean values for the main categories of estimated costs and outcomes of interest, as well as mean differences between the comparator groups. If applicable, report incremental cost-effectiveness ratios.	____________
Characterizing uncertainty	20a	*Single study–based economic evaluation*: Describe the effects of sampling uncertainty for estimated incremental cost, incremental effectiveness, and incremental cost-effectiveness, together with the impact of methodological assumptions (such as discount rate, study perspective).	____________
	20b	*Model-based economic evaluation*: Describe the effects on the results of uncertainty for all input parameters, and uncertainty related to the structure of the model and assumptions.	____________
Characterizing heterogeneity	21	If applicable, report differences in costs, outcomes, or cost-effectiveness that can be explained by variations between subgroups of patients with different baseline characteristics or other observed variability in effects that are not reducible by more information.	____________
**Discussion**			
Study findings, limitations, generalizability, and current knowledge	22	Summarize key study findings and describe how they support the conclusions reached. Discuss limitations and the generalizability of the findings and how the findings fit with current knowledge.	____________
Other			
Source of funding	23	Describe how the study was funded and the role of the funder in the identification, design, conduct, and reporting of the analysis. Describe other nonmonetary sources of support.	____________
Conflicts of interest	24	Describe any potential for conflict of interest among study contributors in accordance with journal policy. In the absence of a journal policy, we recommend authors comply with International Committee of Medical Journal Editors' recommendations.	____________

Note. For consistency, the CHEERS statement checklist format is based on the format of the CONSORT statement checklist.

Source: [3], table 2, pages 235, 236
